# Head Trauma Mechanisms and Neuroimaging Findings in Psychiatric Inpatients Referred for Neurosurgical Consultation

**DOI:** 10.3390/diagnostics16142265

**Published:** 2026-07-20

**Authors:** Ilhan Aydin, Efecan Cekic, Berkay Kef, Asya Gokceli, Egemen Gok, Orhun Mete Cevik, Ahmet Oz, Melih Ucer, Ozden Erhan Sofuoglu, Ceyhan Oflezer, Erhan Emel, Murad Asilturk, Sahin Hanalioglu

**Affiliations:** 1 Department of Neurosurgery, Istanbul Bakırköy Prof. Dr. Mazhar Osman Training and Research Hospital for Mental Health and Neurological Disorders, 34147 Istanbul, Türkiye; asyagokceli.ag@gmail.com (A.G.); orhunmc@gmail.com (O.M.C.); erhansofu@hotmail.com (O.E.S.);; 2 Department of Neurosurgery, Faculty of Medicine, Hacettepe University, 06100 Ankara, Türkiye; efecancekic@hacettepe.edu.tr (E.C.); egemen4552@gmail.com (E.G.); hanalioglu@hacettepe.edu.tr (S.H.); 3 Department of Neurosurgery, Biruni University Hospital, 34295 Istanbul, Türkiye; bkef@biruni.edu.tr (B.K.); mucer@biruni.edu.tr (M.U.); 4 Department of Radiology, Istanbul Bakırköy Prof. Dr. Mazhar Osman Training and Research Hospital for Mental Health and Neurological Disorders, 34147 Istanbul, Türkiye; ahmet.oz@istanbul.edu.tr; 5 Department of Anesthesiology and Reanimation, Istanbul Bakırköy Prof. Dr. Mazhar Osman Training and Research Hospital for Mental Health and Neurological Disorders, 34147 Istanbul, Türkiye; coflezer@yahoo.com; 6 Department of Neurosurgery, Medicana Ataköy Hospital, 34158 Istanbul, Türkiye; muradasil@gmail.com

**Keywords:** head trauma, psychiatric inpatients, neurosurgical consultation, neuroimaging, trauma mechanisms, self-harm, assault

## Abstract

**Background**: Psychiatric inpatients may be vulnerable to head trauma; however, associations among psychiatric diagnosis, trauma mechanisms, and acute trauma-related neuroimaging findings among patients requiring neurosurgical consultation remain unclear. **Methods**: This retrospective observational study included psychiatric inpatients referred for neurosurgical consultation after head trauma at a tertiary neuropsychiatric specialty hospital between July 2022 and December 2025. Psychiatric diagnoses were grouped into psychotic, mood, substance use, organic/symptomatic, and other disorders. Trauma mechanisms were classified as falls, self-harm, assault/fight-related injuries, and other trauma types. Radiological findings were classified as trauma-related abnormalities or secondary incidental findings, including age-related changes. Associations were evaluated using chi-square tests, with false discovery rate correction applied to global and binary comparisons. Exploratory multivariable regression models were used as sensitivity analyses. **Results**: The cohort comprised 461 psychiatric inpatients who sustained head trauma, underwent radiological imaging, and were referred for neurosurgical consultation. Trauma mechanisms differed significantly across psychiatric diagnostic groups (χ^2^ = 30.89, *p* = 0.002). Falls were the most common mechanism (189/461, 41.0%). Assault/fight-related trauma showed the clearest diagnosis-associated pattern (χ^2^ = 19.72, *p* < 0.001, Cramér’s V = 0.207) and remained the only significant binary outcome after false discovery rate correction. Acute trauma-related imaging findings were observed in 35 patients (7.6%) and were not associated with psychiatric diagnosis (χ^2^ = 6.16, *p* = 0.187). Calcifications were the most frequent imaging finding (191/461, 41.4%). **Conclusions**: In psychiatric inpatients, while head trauma patterns and neuroimaging findings have the potential to be explained by psychiatric diagnoses, they may also reflect unmeasured behavioral and clinical factors.

## 1. Introduction

Patients with psychiatric disorders represent a vulnerable population in whom medical and neurological comorbidities frequently coexist and who may have an increased risk of head trauma due to both their underlying conditions and the effects of psychotropic medications [1]. Head trauma is a clinically relevant complication in this group and often necessitates neurosurgical consultation to exclude or manage intracranial pathology [2]. In large psychiatric institutions, such consultations are not uncommon and may represent a substantial clinical burden. Despite this, the clinical and radiological characteristics of head trauma in psychiatric inpatient populations remain insufficiently defined.

Several factors contribute to the increased risk of head trauma in psychiatric patients. Impulsivity and impaired judgement play a central role [3]. Psychotropic medications may further increase vulnerability by impairing balance, alertness, and cognitive function. Substance use is another well-established contributor [4]. Self-harm behaviors are particularly frequent in patients with mood and psychotic disorders [5]. In addition, older patients are more prone to falls due to cognitive decline, reduced mobility, and medication-related side effects [6,7]. However, whether psychiatric diagnostic groups show distinct trauma patterns in inpatient settings remains unclear.

Neuroimaging plays a central role in evaluating head trauma, particularly in detecting acute intracranial pathology and guiding clinical management [8,9]. Its primary purpose is the detection of acute intracranial lesions, such as hemorrhage, contusion, pneumocephalus, or mass effect [2,10,11]. However, imaging frequently reveals incidental findings, including neurodevelopmental variants and age-related atrophy. Earlier studies suggested potential associations between structural brain abnormalities and psychiatric disorders, particularly in schizophrenia-spectrum conditions [12]. More recent evidence indicates that these findings lack diagnostic specificity and may reflect broader neurodevelopmental or degenerative processes [13,14,15]. Distinguishing acute trauma-related lesions from secondary or incidental findings is therefore important when evaluating neuroimaging patterns in psychiatric inpatients.

In this context, the present study aimed to evaluate how trauma mechanisms differ across psychiatric diagnostic groups in a large cohort of psychiatric inpatients referred for neurosurgical consultation following head trauma. We also examined the association between psychiatric diagnosis and neuroimaging findings, considering acute trauma-related findings separately from secondary or incidental abnormalities. In addition, demographic correlates, particularly age, were explored in relation to trauma mechanisms and radiological findings.

## 2. Methods

### 2.1. Study Design

This retrospective observational study included psychiatric inpatients who underwent neurosurgical consultation following head trauma at a high-volume tertiary psychiatric hospital between July 2022 and December 2025. The institution is one of the largest psychiatric specialty hospitals in Türkiye and Europe, with a history of more than 100 years and a capacity of approximately 1333 beds. The institution does not function as a general emergency department or general trauma center; neurosurgical care is provided mainly for neurosurgical emergencies and inpatient consultations from psychiatry, neurology, and related services. Therefore, the study population consisted of psychiatric inpatients with head trauma who were referred for neurosurgical evaluation rather than all psychiatric inpatients who sustained head trauma during the study period.

According to institutional clinical workflow, psychiatric inpatients referred for neurosurgical consultation after head trauma undergo cranial CT. In addition, routine cranial MRI is available as part of the psychiatric inpatient assessment protocol to exclude underlying intracranial organic pathology. Therefore, complete CT and MRI data were available for all included patients, and no patient was excluded because of missing neuroimaging data after cohort identification.

Relevant data from consecutive patients were collected from the institutional electronic medical records. The study protocol was approved by the Biruni University Clinical Research Ethics Committee before retrospective data collection and analysis were initiated (Date: 23 February 2026; No: 2024-BİAEK/18-79), and all data were anonymized before analysis. Patients provide institutional consent for routine clinical evaluation and procedures. As this study used anonymized clinical and radiological data collected during routine care, no additional procedures, interventions, or patient contact were performed, and separate study-specific informed consent was not obtained.

### 2.2. Clinical Variables and Diagnostic Grouping

Demographic variables included age and sex. Medication exposure was retrieved from medical documents and were classified as antipsychotics, mood stabilizers, antidepressants, benzodiazepines, or sedative/hypnotics. Psychiatric diagnoses were classified from Diagnostic and Statistical Manual of Mental Disorders (DSM)-based clinical records and grouped into five categories as psychotic disorders (schizophrenia and schizoaffective disorder); mood disorders (bipolar disorder and depressive disorder); substance use disorders; organic/symptomatic disorders (organic or symptomatic psychiatric conditions); and other psychiatric disorders (including acute non-specific psychiatric presentations, adjustment and personality disorders, and intellectual disability). Psychiatric diagnoses were grouped into broader DSM-based clinical domains to balance clinical interpretability and statistical stability. Less frequent diagnostic categories were combined as “other psychiatric disorders” to avoid very small analytical cells.

Trauma mechanisms were initially recorded as assault/fight, falls, blunt trauma, seizure-related trauma, self-injurious behavior, suicide attempt, syncope/dizziness-related fall, and traffic accidents. For inferential analyses, trauma mechanisms were consolidated into four groups as falls (including falls and syncope/dizziness-related falls), self-harm (including self-injurious behavior and suicide attempts), assault/fight-related injuries, and other trauma types (including blunt trauma, seizure-related trauma, and traffic accidents).

### 2.3. Neuroimaging Assessment

All included patients had both cranial CT and MRI available; no patient had CT-only or MRI-only imaging. Imaging findings were based on official radiology reports and specialist radiological evaluation. Radiological findings were grouped into five clinically meaningful categories as normal/non-specific findings (non-specific imaging findings); calcifications (pineal/choroid plexus calcification and falx calcification); CSF-space/developmental variants (partial empty sella, arachnoid cyst/cystic lesion, and Type I Chiari malformation); structural/parenchymal findings (hydrocephalus, infarct/CVA, and structural lesions); and trauma-related findings (contusion, fracture, hematoma/hemorrhage, and scalp edema/laceration). Acute trauma-related imaging findings were defined as the primary imaging outcome, whereas calcifications, CSF-space/developmental variants, and structural/parenchymal findings were treated as secondary or incidental imaging findings.

### 2.4. Statistical Analysis

Statistical analyses were performed using R (version 4.3.3, R Foundation for Statistical Computing, Vienna, Austria). Categorical variables were summarized as frequencies and percentages, and continuous variables as medians with interquartile ranges. Associations between psychiatric diagnosis, trauma mechanisms, and radiological findings were analyzed using the chi-square test. Effect size was quantified with Cramér’s V. Standardized residuals were used as exploratory cell-level diagnostics for heatmap visualization.

In addition, selected binary outcomes were examined for falls, self-harm, assault/fight-related injuries, and acute trauma-related imaging findings. Secondary binary analyses were considered exploratory. Because multiple secondary analyses were performed, Benjamini–Hochberg false discovery rate correction was applied to the main global and binary chi-square tests.

Exploratory multivariable binary logistic regression models were constructed for assault/fight-related injuries and acute trauma-related imaging findings. For the assault/fight model, age, sex, psychiatric diagnostic group, medical comorbidity, and psychotropic medication status were included as covariates. Adjusted odds ratios (ORs) with 95% confidence intervals (CIs) were calculated. Age-adjusted logistic regression models were performed for key secondary imaging outcomes, including calcifications and structural/parenchymal findings.

Post hoc sensitivity analyses were performed to evaluate the heterogeneity of the other psychiatric disorders group, which included acute non-specific psychiatric presentations, adjustment/personality disorders, and intellectual disability. First, these three subgroups were entered separately into the multivariable logistic regression model for assault/fight-related trauma. Second, the primary model was repeated after excluding each subgroup in turn. These analyses were considered exploratory due to the small numbers of patients and events within individual subgroups. Logistic regression and subgroup analyses were considered exploratory. A two-sided *p*-value of less than 0.05 was considered statistically significant.

## 3. Results

### 3.1. Demographic and Clinical Characteristics

During the study period, 49,532 patients received inpatient psychiatric treatment. Among these, 461 psychiatric inpatients sustained head trauma, underwent radiological imaging, and were referred for neurosurgical consultation. All 461 patients had both CT and MRI data available. Therefore, imaging modality did not differ across psychiatric diagnostic groups. No patient was excluded because of missing neuroimaging data.

The median age was 43.0 years (interquartile range 30.0–55.0), and 282 patients (61.2%) were male. Psychotropic medication use was present in 299 patients (64.9%), and 200 patients (43.4%) had at least one documented medical comorbidity. In the available medication records, antipsychotics were the most frequent class (n = 208), followed by mood stabilizers (n = 95), antidepressants (n = 38), benzodiazepines (n = 22), and sedative/hypnotics (n = 3).

The largest diagnostic groups were schizophrenia/schizoaffective disorder (n = 103, 22.3%), bipolar disorder (n = 102, 22.1%), and organic/symptomatic disorders (n = 98, 21.3%). Patients with schizophrenia-spectrum disorders were older, whereas those with adjustment/personality disorders were younger. Male predominance was greatest in substance use disorders and acute non-specific psychiatric presentations (Table 1). Additionally, 10 patients had radiologically evident traumatic brain injury (hematoma/hemorrhage = 7, contusion = 3).

### 3.2. Trauma Mechanisms Across Psychiatric Diagnoses

**Figure 1:**
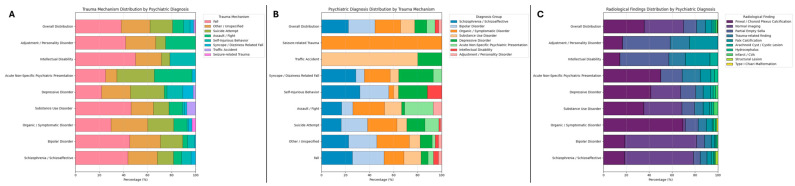
Distribution of trauma mechanisms, psychiatric diagnoses, and radiological findings across study groups. (**A**) Proportional distribution of trauma mechanisms across psychiatric diagnostic categories. Trauma mechanisms are presented as percentages within each diagnosis group. (**B**) Distribution of psychiatric diagnoses across trauma mechanisms, illustrating the relative contribution of each diagnostic category within specific trauma types. (**C**) Distribution of radiological findings across psychiatric diagnostic groups, including incidental, non-specific, and structural abnormalities. All panels display normalized percentages (100% stacked bar plots), allowing comparison of relative distributions across groups. Overall distribution represents the aggregate proportion of each category in the full cohort. Radiological findings include both acute trauma-related findings and secondary/incidental findings.

Trauma mechanisms differed significantly across psychiatric diagnostic groups overall (χ^2^ = 30.89, *p* = 0.002), although the effect size was small (Cramér’s V = 0.150). Fall-related trauma was the most common mechanism overall (189/461, 41.0%), particularly in mood disorders (61/148, 41.2%), psychotic disorders (49/103, 47.6%), and substance use disorders (26/54, 48.1%) (Figure 1). Detailed distributions of individual radiological findings and their associated demographic and clinical characteristics are provided in Table 2. Organic/symptomatic disorders showed a comparatively lower proportion of fall-related trauma (32/98, 32.7%), with a more even distribution across other mechanisms. However, fall-related trauma did not differ significantly across diagnostic groups in binary analysis (χ^2^ = 6.36, *p* = 0.174, Cramér’s V = 0.117).

Other trauma mechanisms were relatively frequent in organic/symptomatic disorders (33/98, 33.7%) compared to mood (38/148, 25.7%) and psychotic disorders (25/103, 24.3%). Despite these differences, the distribution of other trauma mechanisms did not reach statistical significance in binary analysis (χ^2^ = 6.50, *p* = 0.165, Cramér’s V = 0.119).

Self-harm-related presentations were most common in mood disorders (44/148, 29.7%), followed by organic/symptomatic (22/98, 22.4%) and psychotic disorders (22/103, 21.4%), while remaining less frequent in substance use disorders (8/54, 14.8%). The distribution of self-harm across diagnostic groups was not statistically significant (χ^2^ = 5.78, *p* = 0.216, Cramér’s V = 0.112).

**Figure 2:**
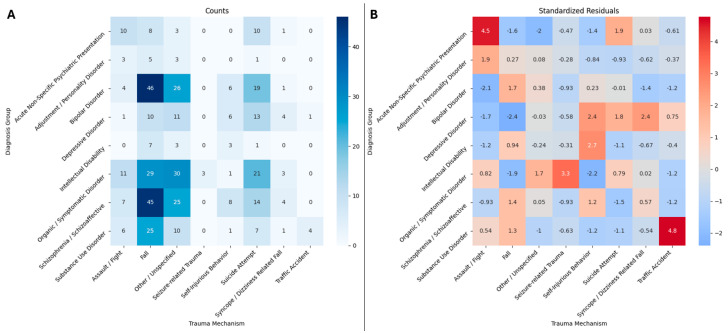
Association between psychiatric diagnostic groups and trauma mechanisms. (**A**) Heatmap showing the absolute counts of trauma mechanisms across psychiatric diagnostic categories. (**B**) Heatmap of standardized residuals derived from the chi-square analysis of the same contingency table. Standardized residuals represent the deviation of observed counts from expected counts under the assumption of independence between variables, scaled by the standard error. Positive residuals indicate that a given trauma mechanism occurs more frequently than expected within a diagnostic group, whereas negative residuals indicate under-representation. All trauma mechanisms and psychiatric diagnostic categories are presented without subgrouping to preserve the full structure of the contingency table. Standardized residuals were used as an exploratory measure to identify cell-level deviations from expected frequencies.

In contrast, assault/fight-related injuries showed the clearest diagnosis-linked pattern (χ^2^ = 19.72, *p* < 0.001, Cramér’s V = 0.207). Assault and fighting were proportionally most frequent in the other diagnoses group, including acute non-specific psychiatric presentations, adjustment/personality disorders, and intellectual disability (13/58, 22.4%), compared to substantially lower rates in mood (5/148, 3.4%), psychotic (7/103, 6.8%), organic (11/98, 11.2%), and substance use disorders (6/54, 11.1%). The distribution of trauma mechanisms across psychiatric diagnoses, including frequencies and standardized residuals, is presented in Figure 2 without grouping.

**Figure 3:**
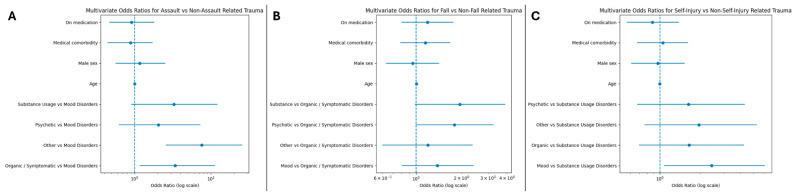
Multivariable Logistic Regression Analyses of Trauma Mechanisms by Psychiatric Diagnosis. Forest plots displaying adjusted odds ratios (ORs) with 95% confidence intervals (CIs) for factors associated with (**A**) assault/fight-related trauma, (**B**) fall-related trauma, and (**C**) self-injury-related trauma. Each model was adjusted for age, sex, medical comorbidity, and psychotropic medication status. Psychiatric diagnostic groups were included as categorical variables, with mood disorders serving as the reference category in (**A**), organic/symptomatic disorders in (**B**), and substance use disorders in (**C**). The vertical dashed line represents an OR of 1.0 (no association). Points indicate estimated ORs and horizontal bars represent 95% CIs.

In exploratory multivariable logistic regression, assault/fight-related injury remained independently associated with psychiatric diagnosis. Compared with mood disorders, the odds were higher in organic/symptomatic disorders (OR 3.39, 95% CI 1.17–11.25, *p* = 0.031) and in other psychiatric disorders (OR 7.57, 95% CI 2.58–25.54, *p* < 0.001). In terms of self-injury related trauma, patients with mood disorders had higher odds compared to substance use disorders (OR 2.43, 95% CI 1.08–6.09, *p* = 0.042). No independent associations were observed between other trauma types and diagnostic groups, nor with age, sex, substance use disorders, medical comorbidity, or medication status (Figure 3).

After false discovery rate correction, the global association between psychiatric diagnosis and trauma mechanism remained significant, and assault/fight-related trauma remained the only significant binary trauma outcome. Fall-related, self-harm-related, and other trauma mechanisms were not significant after correction.

In post hoc sensitivity analyses of the “other psychiatric disorders” group, assault/fight-related trauma occurred in 10 of 32 patients with acute non-specific psychiatric presentations (31.2%), 3 of 12 patients with adjustment/personality disorders (25.0%), and 0 of 14 patients with intellectual disability (0.0%). When these subgroups were separated in the multivariable model, acute non-specific psychiatric presentations and adjustment/personality disorders showed higher odds of assault/fight-related trauma compared with mood disorders, whereas intellectual disability had no assault/fight-related events, making the estimate unstable and not clinically interpretable.

In leave-one-subgroup-out analyses, the association between the remaining “other psychiatric disorders” group and assault/fight-related trauma remained significant after excluding adjustment/personality disorders (OR 7.42, 95% CI 2.42–25.63, *p* < 0.001) and after excluding intellectual disability (OR 11.77, 95% CI 3.86–41.24, *p* < 0.001). After excluding acute non-specific psychiatric presentations, the association was attenuated and no longer statistically significant (OR 3.10, 95% CI 0.58–14.18, *p* = 0.151). These findings suggest that the assault/fight-related trauma signal within the heterogeneous “other psychiatric disorders” group was driven mainly by acute non-specific psychiatric presentations and partly by adjustment/personality disorders, rather than by intellectual disability (Table 3).

### 3.3. Imaging Findings Across Psychiatric Diagnoses

**Figure 4:**
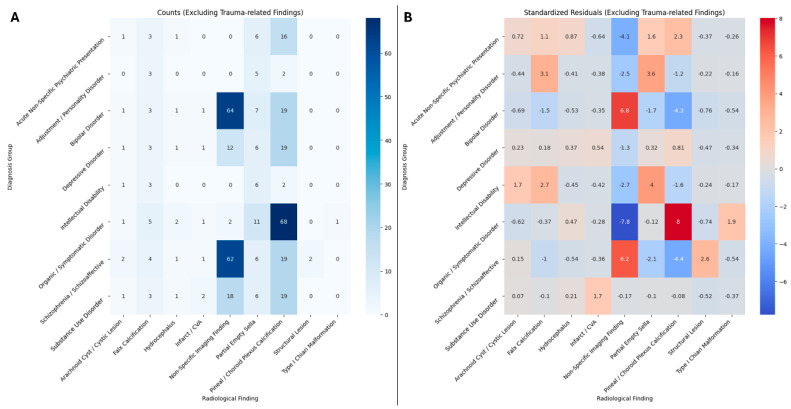
Association between psychiatric diagnostic groups and radiological findings, excluding trauma-related findings. (**A**) Heatmap showing the absolute counts of radiological findings across psychiatric diagnostic categories. (**B**) Heatmap of standardized residuals derived from the chi-square analysis of the same contingency table. Standardized residuals represent the deviation of observed counts from expected counts under the assumption of independence between variables, scaled by the standard error. Positive residuals indicate that a given radiological finding occurs more frequently than expected within a diagnostic group, whereas negative residuals indicate under-representation. All radiological findings and psychiatric diagnostic categories are presented without subgrouping to preserve the full structure of the contingency table. Standardized residuals were used as an exploratory measure to identify cell-level deviations from expected frequencies. Acute trauma-related findings were excluded from this analysis to show the distribution of secondary/incidental radiological findings across psychiatric diagnostic groups.

Radiological findings differed significantly across psychiatric diagnostic groups overall (χ^2^ = 150.27, df = 16, *p* < 0.001), with a moderate effect size (Cramér’s V = 0.286). Calcifications were frequent but were analyzed as secondary/incidental findings rather than acute trauma-related abnormalities. Calcifications were the most frequent imaging finding (191/461, 41.4%), followed by normal/non-specific findings (158/461, 34.3%), CSF-space/developmental variants (62/461, 13.4%), acute trauma-related findings (35/461, 7.6%), and structural/parenchymal findings (15/461, 3.3%) (Figure 1). Calcifications were particularly prominent in organic/symptomatic disorders and were strongly associated with psychiatric diagnosis (χ^2^ = 69.74, *p* < 0.001, Cramér’s V = 0.389) (Figure 4). Congenital/developmental variants also differed significantly across groups (χ^2^ = 22.85, *p* < 0.001, Cramér’s V = 0.223), with relative over-representation in the other psychiatric disorders group. By contrast, acute trauma-related imaging findings were not significantly associated with psychiatric diagnosis (χ^2^ = 6.16, *p* = 0.187, Cramér’s V = 0.116).

After false discovery rate correction, the global association between psychiatric diagnosis and imaging category remained significant, whereas acute trauma-related imaging findings were not significantly associated with psychiatric diagnosis. In age-adjusted analyses, calcification was associated with age (OR 1.02 per year, 95% CI 1.01–1.04, *p* = 0.002) and remained more frequent in the organic/symptomatic group compared with mood disorders (OR 7.65, 95% CI 4.31–14.00, *p* < 0.001). Structural/parenchymal findings were not significantly associated with age or diagnostic group after adjustment for age.

## 4. Discussion

### 4.1. Principal Findings and Interpretation

This study examined trauma mechanisms and neuroimaging findings in psychiatric inpatients referred for neurosurgical consultation after head trauma. Trauma mechanisms differed significantly across psychiatric diagnostic groups overall, but the effect size was small, suggesting that diagnosis alone explained only a limited part of trauma pattern variability. This was supported by the binary analyses: neither falls nor self-harm showed a significant association with diagnosis. Assault/fight-related trauma was the main exception. It showed the clearest diagnosis-linked pattern, with the highest relative frequency in the other psychiatric disorders group (non-specific psychiatric presentations, adjustment/personality disorders, and intellectual disability), and this association persisted after adjustment for age, sex, substance use disorders, medical comorbidity, and medication status. The sensitivity analyses indicated that this association was driven mainly by acute non-specific psychiatric presentations and, to a lesser extent, adjustment/personality disorders, whereas intellectual disability did not contribute assault/fight-related events.

A similar pattern emerged in the imaging results. Radiological categories differed significantly across psychiatric groups, with a moderate overall effect size, but this pattern was driven mainly by incidental and nonspecific findings rather than acute trauma-related lesions. Calcifications and congenital/developmental variants accounted for most of the between-group variation. Importantly, although our findings suggest an association between psychiatric diagnoses and trauma patterns, we believe that these relationships may also be influenced by factors not captured in the present study, including patients’ acute clinical presentations, detailed comorbidity profiles, and medication histories.

### 4.2. Trauma Patterns and Behavioral Determinants

Our results suggest that psychiatric diagnosis alone may not fully explain trauma risk in psychiatric inpatients. Falls made up 41.0% of all trauma events in our cohort. That made them the most common mechanism overall. Even so, fall-related trauma did not differ significantly across diagnostic groups in binary analysis. This is in line with earlier hospital-based studies showing that falls are common in inpatient populations and are usually linked to age, physical vulnerability, medication burden, and reduced mobility rather than to one specific psychiatric disorder [16].

Assault-related trauma showed the clearest diagnosis-linked pattern in our data. It was proportionally most frequent in the other diagnoses group, which included acute non-specific psychiatric presentations, adjustment/personality disorders, and intellectual disability. This pattern may reflect factors such as acute agitation, interpersonal conflict, poor impulse control, and unstable behavioral states, although these factors were not directly measured in the present study. This interpretation is also supported by population-based studies. In the meta-analysis by Fazel et al., 9.9% of patients with schizophrenia and related psychoses had a violent outcome compared with 1.6% of general-population controls [17]. In the Swedish population cohort by Fazel et al., 13.2% of patients with schizophrenia had at least one violent offense compared with 5.3% of matched controls. More importantly, that excess risk was heavily influenced by substance use disorders. Among patients with schizophrenia and substance use disorders comorbidity, 27.6% had a violent offense, whereas the corresponding figure was 8.5% in those without substance use disorders [18]. That pattern is highly relevant to our cohort. In our data too, assault-related trauma clustered in groups characterized by greater behavioral instability. The sensitivity analyses further clarify the interpretation of the “other psychiatric disorders” group. The higher proportion of assault/fight-related trauma was not uniformly distributed across all components of this heterogeneous group. Instead, the signal appeared to be driven mainly by acute non-specific psychiatric presentations and adjustment/personality disorders, whereas intellectual disability did not contribute assault/fight-related events. This supports the interpretation that acute behavioral dysregulation, interpersonal conflict, and unstable clinical states may be more relevant to assault/fight-related trauma than diagnostic grouping alone. These subgroup-level findings should not be interpreted as definitive evidence of diagnosis-specific risk, particularly for small diagnostic categories.

Self-harm followed a somewhat different pattern. In our cohort, it was numerically more frequent in mood disorders and organic/symptomatic disorders, but the difference between groups was not statistically significant. This does not necessarily contradict the broader self-harm literature. Rather, it likely reflects the way our cohort was selected. We studied psychiatric inpatients who had already sustained head trauma serious enough to prompt neurosurgical consultation. In broader hospital-based self-harm populations, psychiatric disorders are extremely common, with depression, anxiety, and alcohol misuse among the most frequent diagnoses [5]. Our data suggest that once the sample is narrowed to psychiatric inpatients with trauma and neurosurgical referral, diagnostic differences in self-harm may become less distinct than expected (Table 4).

Overall, these findings suggest that diagnosis alone may be insufficient to explain trauma patterns in psychiatric inpatients.

### 4.3. Neuroimaging Findings and Their Clinical Relevance

One of the most important findings of this study is that neuroimaging abnormalities were common, but most were not diagnosis-specific. In our cohort, calcifications were the most frequent imaging finding overall and were particularly common in organic/symptomatic disorders. Congenital findings also varied significantly across groups. However, acute trauma-related abnormalities did not show a significant association with psychiatric diagnosis. This distinction matters.

Physiological and age-related calcifications are very common on CT, even in people without psychiatric illness. In the CT study by Yalcin et al., pineal calcifications were seen in 71.6% of subjects, choroid plexus calcifications in 70.2%, dural calcifications in 12.5%, vascular calcifications in 3.5%, and basal ganglia calcifications in 1.3% [19]. Because pineal, choroid plexus, and falx calcifications are commonly physiological or age-related, their high frequency should not be interpreted as evidence of diagnosis-specific traumatic or psychiatric pathology. The clinically relevant primary imaging outcome was acute trauma-related pathology, which was uncommon and not significantly associated with psychiatric diagnosis. In age-adjusted analyses, calcification remained associated with age and was more frequent in the organic/symptomatic group compared with mood disorders; however, this finding should still be interpreted cautiously because calcifications are commonly physiological or age-related incidental findings. Structural/parenchymal findings were uncommon and showed no significant association with age or psychiatric diagnostic group after age adjustment.

The same applies to congenital variants. Earlier structural imaging literature, especially in schizophrenia, raised the possibility that some neurodevelopmental variants might be more common in psychotic disorders [12]. More recent large-scale imaging studies have made that picture much less specific. In the ENIGMA schizophrenia consortium, which included 4474 patients and 5098 controls, cortical differences were detectable at the group level, but the effect sizes were modest and broadly distributed rather than diagnostically specific [20]. That is very much in line with our findings. Congenital and incidental abnormalities contributed to group-level radiological differences, but they did not provide a meaningful explanation for trauma mechanisms and should not be overinterpreted as markers of psychiatric disease in the context of head trauma.

The absence of a relationship between acute trauma-related imaging findings and psychiatric diagnosis is probably the most clinically useful imaging result in this study. It suggests that lesions such as hemorrhage, contusion, pneumocephalus, or mass effect are determined primarily by injury biomechanics and severity rather than by psychiatric diagnosis. This is consistent with the wider neurotrauma literature, where CT and MRI are used mainly to identify surgically relevant pathology and to estimate injury burden rather than to explain psychiatric mechanisms [21]. In practical terms, our findings support a straightforward interpretation: structural imaging is essential in psychiatric inpatients with head trauma because it identifies acute neurosurgical pathology, but it offers limited value for distinguishing between psychiatric diagnostic categories.

### 4.4. Clinical Implications

These findings have several practical implications. First, trauma prevention in psychiatric inpatient units should focus on modifiable risk domains rather than on diagnosis alone. Fall prevention measures in older patients, close observation during periods of agitation, and active management of substance use disorders are likely to be more useful than diagnosis-based risk labeling.

Second, neuroimaging in this setting needs to be interpreted carefully. Incidental findings are common. They can contribute to statistically significant group differences, but they are often clinically nonspecific. Without proper context, benign developmental variants or age-related changes may be mistaken for meaningful pathology.

Third, the assault signal in the acute and non-specific diagnostic subgroup suggests that diagnosis alone may not fully explain assault/fight-related trauma and that unmeasured dynamic clinical factors may also contribute. This has direct relevance to inpatient monitoring and communication among psychiatry, emergency care, radiology, and neurosurgery.

### 4.5. Limitations and Future Directions

This study has several limitations. Its retrospective, single-center design limits causal interpretation and introduces the possibility of selection and documentation bias. The cohort represents psychiatric inpatients with head trauma who required neurosurgical consultation, rather than all psychiatric patients with head trauma. The absence of a non-psychiatric control group with comparable head trauma also limits direct comparative interpretation.

The study was not powered for definitive comparisons across small diagnostic subgroups; therefore, subgroup-level findings should be considered exploratory. Although sensitivity analyses were performed for the components of the heterogeneous “other psychiatric disorders” group, the subgroup sizes were small, particularly for adjustment/personality disorders and intellectual disability. Therefore, these findings should be interpreted as exploratory and hypothesis-generating rather than definitive subgroup-level evidence. Moreover, owing to the inherent limitations of the retrospective study design, data on patients’ acute behavioral states at the time of psychiatric presentation were unavailable for analysis.

Future studies should ideally use prospective, multicenter designs with larger and more balanced subgroup distributions and more detailed assessment of trauma circumstances, medication exposure, acute behavioral status, and neurological comorbidity.

## 5. Conclusions

In this large, single-center cohort of psychiatric inpatients referred for neurosurgical consultation after head trauma, falls were the most common trauma mechanism, while assault/fight-related trauma showed the clearest diagnosis-associated pattern. Acute trauma-related imaging findings were uncommon and were not significantly associated with psychiatric diagnosis, whereas much of the overall radiological variation was driven by secondary or incidental findings such as calcifications and CSF-space/developmental variants. Although our analyses suggested potential associations among trauma mechanisms, psychiatric diagnoses, and imaging findings, these relationships could not be fully characterized because of the exploratory nature of the analyses and the possible influence of unmeasured behavioral factors at presentation, warranting further investigation in future studies.

## Figures and Tables

**Table 1:** Baseline characteristics of patients stratified by psychiatric diagnosis.

Psychiatric Diagnosis	Age (Median [Q1–Q3])	Male, n (%)	On Medication (Yes, n [%])	Medical Comorbidity (Yes, n [%])
Schizophrenia/Schizoaffective (n = 103)	52.0 (43.0–63.5)	60 (58.3%)	63 (61.2%)	44 (42.7%)
Bipolar Disorder (n = 102)	44.0 (33.0–54.8)	36 (35.3%)	72 (70.6%)	51 (50.0%)
Organic/Symptomatic Disorder (n = 98)	39.5 (28.0–53.0)	66 (67.3%)	62 (63.3%)	38 (38.8%)
Substance Use Disorder (n = 54)	35.0 (28.2–50.2)	46 (85.2%)	36 (66.7%)	19 (35.2%)
Depressive Disorder (n = 46)	35.0 (27.0–53.0)	29 (63.0%)	29 (63.0%)	25 (54.3%)
Acute Non-Specific Psychiatric Presentation (n = 32)	35.0 (30.0–48.5)	26 (81.2%)	22 (68.8%)	13 (40.6%)
Intellectual Disability (n = 14)	33.0 (24.0–38.5)	7 (50.0%)	7 (50.0%)	6 (42.9%)
Adjustment/Personality Disorder (n = 12)	22.0 (21.0–27.5)	12 (100.0%)	8 (66.7%)	4 (33.3%)

**Table 2:** Distribution of radiological findings and associated demographic and clinical characteristics.

Radiological Findings	Age (Median [Q1–Q3])	Male, n (%)	On Medication (Yes, n [%])	Medical Comorbidity (Yes, n [%])
Arachnoid Cyst/Cystic Lesion (8 [1.7%])	33 (25.5–36.5)	4 [50%]	3 [37.5%]	3 [37.5%]
Contusion (3 [0.7%])	40 (32.5–43)	1 [33.3%]	1 [33.3%]	2 [66.7%]
Falx Calcification (27 [5.9%])	50 (35.5–61.5)	17 [63%]	17 [63%]	11 [40.7%]
Fracture (20 [4.3%])	41.5 (30.8–60.5)	15 [75%]	16 [80%]	13 [65%]
Hematoma/Hemorrhage (7 [1.5%])	44 (34–51.5)	5 [71.4%]	4 [57.1%]	2 [28.6%]
Hydrocephalus (7 [1.5%])	37 (36–57.5)	5 [71.4%]	4 [57.1%]	3 [42.9%]
Infarct/CVA (6 [1.3%])	50 (45–58.8)	6 [100%]	3 [50%]	3 [50%]
Non-Specific Imaging Finding (158 [34.3%])	42 (28–51.8)	94 [59.5%]	110 [69.6%]	80 [50.6%]
Partial Empty Sella (53 [11.5%])	43 (32–56)	34 [64.2%]	33 [62.3%]	19 [35.8%]
Pineal/Choroid Plexus Calcification (164 [35.6%])	41 (30–54)	96 [58.5%]	105 [64%]	62 [37.8%]
Scalp Edema/Laceration (5 [1.1%])	43 (42–54)	3 [60%]	2 [40%]	2 [40%]
Structural Lesion (2 [0.4%])	59.5 (52.8–66.2)	1 [50%]	–	–
Type I Chiari Malformation (1 [0.2%])	48 (48–48)	1 [100%]	1 [100%]	–

Calcifications, CSF-space/developmental variants, and structural/parenchymal abnormalities were considered secondary or incidental imaging findings. Acute trauma-related findings included contusion, fracture, hematoma/hemorrhage, and scalp edema/laceration.

**Table 3:** Association Between Psychiatric Diagnosis and Assault- and Fall-Related Trauma. Multivariable logistic regression analyses evaluating factors associated with assault- and fall-related trauma. For the assault model, mood disorders were used as the reference category; for the fall model, organic/symptomatic disorders were used as the reference category. Odds ratios (ORs) with 95% confidence intervals (CIs) are presented. Both models were adjusted for age, sex, medical comorbidity, and medication status.

Assault vs. Non-Assault Related Trauma Types			Fall vs. Non-Fall Related Trauma Types		
Variable	OR (95% CI)	*p*-Value	Variable	OR (95% CI)	*p*-Value
Organic/Symptomatic vs. Mood Disorders	3.39 (1.17–11.25)	0.031	Mood vs. Organic/Symptomatic Disorders	1.38 (0.80–2.40)	0.245
Other vs. Mood Disorders	7.57 (2.58–25.54)	<0.001	Other vs. Organic/Symptomatic Disorders	1.20 (0.60–2.37)	0.611
Psychotic vs. Mood Disorders	2.05 (0.62–7.27)	0.242	Psychotic vs. Organic/Symptomatic Disorders	1.80 (1.00–3.27)	0.053
Substance Usage vs. Mood Disorders	3.25 (0.90–12.23)	0.070	Substance Usage vs. Organic/Symptomatic Disorders	1.95 (0.98–3.89)	0.057
Age	1.00 (0.98–1.02)	0.829	Age	1.00 (0.99–1.02)	0.617
Male sex	1.17 (0.57–2.52)	0.682	Male sex	0.95 (0.63–1.42)	0.796
Medical comorbidity	0.88 (0.44–1.72)	0.716	Medical comorbidity	1.15 (0.78–1.68)	0.476
On medication	0.91 (0.47–1.81)	0.777	On medication	1.19 (0.80–1.77)	0.393

**Table 4:** Association Between Psychiatric Diagnosis and Self-Injury-Related Trauma. Multivariable logistic regression analysis evaluating factors associated with self-injury-related trauma. Substance use disorder was used as the reference category for psychiatric diagnosis. Odds ratios (ORs) with 95% confidence intervals (CIs) are presented. The model was adjusted for age, sex, medical comorbidity, and medication status.

Self-Injury vs. Non-Self-Injury Injury Related Trauma		
Variable	OR (95% CI)	*p*-Value
**Mood vs. Substance Usage Disorders**	**2.43 (1.08–6.09)**	**0.042**
Organic vs. Substance Usage Disorders	1.66 (0.70–4.26)	0.268
Other vs. Substance Usage Disorders	1.95 (0.77–5.30)	0.170
Psychotic vs. Substance Usage Disorders	1.64 (0.68–4.30)	0.291
Age	1.00 (0.98–1.01)	0.485
Male sex	0.96 (0.61–1.53)	0.868
Medical comorbidity	1.05 (0.68–1.62)	0.831
On medication	0.88 (0.56–1.39)	0.577

## Data Availability

The data presented in this study are available on request from the corresponding author.
